# Irish residential heating demand profile dataset

**DOI:** 10.1016/j.dib.2024.111215

**Published:** 2024-12-16

**Authors:** John Curtis, Niall Farrell

**Affiliations:** a Economic and Social Research Institute, Dublin, Ireland; b Trinity College Dublin, Dublin, Ireland; c SFI MaREI Centre for Energy, Climate and Marine, Ireland

**Keywords:** Building energy performance, Residential energy demand, Electrification of heating, Heat pump

## Abstract

This data article describes the operation of gas and oil fuelled residential heating systems in Ireland. Based on almost 10,000 homes, the data presents information on the operation of domestic heating systems (whether turned on/off by the user), and the firing of the boiler during 2-hour slots across a period of two years ending in September 2021 by geographical region. The electrification of heating is government policy, with the ambition that hundreds of thousands of homes will switch from oil and gas fuelled residential heating to heat pumps. Such an outcome will have implications for electricity generation, transmission and distribution, especially during peak demand periods. This dataset can be used as a starting point to examine how additional residential heating loads will impact on the electricity grid and provide insight on where electricity grid strengthening works should be prioritised.

Specifications table

**Table:** 

Subject	Energy (Renewable Energy, Sustainability and the Environment).
Specific subject area	The dataset contains information on residential heating use in Irish homes.
Type of data	The dataset includes information on heating use (on/off) and boiler operation across geographical areas, within 2-hour time slots, for two years.
Data collection	The raw data was collected by smart thermostats situated within individual homes. The data was processed to provide summary data by postal areas.
Data source location	Republic of Ireland.
Data accessibility	Repository name: Mendeley Data… Data identification number: 10.17632/bwt34c5v2z.1 Direct URL to data: http://dx.doi.org/10.17632/bwt34c5v2z.1
Related research article	None.

## 1 Value of the Data

• Research datasets on actual versus synthetic data related to residential heating in Ireland have very small sample sizes (e.g., <50 households with data loggers) versus approx. 10k households underpinning this data. These data include the heterogeneity of heating behaviours that cannot be captured in small N datasets.
• The electrification of heating is government policy, with the ambition that hundreds of thousands of homes will switch from oil and gas fuelled residential heating to heat pumps. This dataset provides time of use of energy demand information that has relevance for the electricity sector that must plan to reliably deliver electricity supplies to match increased electricity loads, including during existing peak electricity load periods.
• Researchers modelling the impact on the electricity grid of additional loads can incorporate this data into their models (capturing spatial and heterogeneity of use variability) to provide insight on where electricity grid strengthening works should be prioritized.

## 2 Background

Research datasets on energy use for residential heating in Ireland collected via data loggers generally comprise small samples (e.g. N <20 [1,2]). Sample sizes are greater for studies based on meter readings (e.g., [[3], [4], [5], [6]]) but low frequency, aggregated data has limited usefulness for understanding residential heating demand profiles. In the absence of large- scale energy-use datasets, synthetic or simulated data based on building archetypes have been developed. For example, Ahern and Norton [7] use data from 35 reference dwellings to characterise over 0.4 million dwellings for the purpose of predicting residential energy consumption, while Ali et al. [8,9] use machine learning approaches to develop synthetic data simulating energy use among one million buildings. A policy ambition is to switch hundreds of thousands of homes to heat pumps, which will have an impact on the electricity grid. Studies modelling heat pump demand profiles are based on small N samples. For example, with data from 19 homes Chesser et al. [10] conclude that their electricity demand profile follows a Gamma distribution. But demand profiles implicit in the smart thermostat data in Meles et al. [11,12] suggests it may be bi-modal. Large-scale datasets such as this can contribute to research residential heating demand.

## 3 Data Description

The dataset comprises two files. CodeBook.csv includes two columns, the first contains variable names of all the variables in the main dataset file (HeatingDemandProfiles.csv), and the second column contains a description of each variable. The file HeatingDemandProfiles.csv contains 11,305 rows including the column title row. Each row comprises data for a 2-hour slot (e.g. 00:00-02:00), in a month (e.g., January), within a postal area (e.g. A63). The row includes the number of households from which the data in that row is drawn, and the number of hourly data observations. The initial dataset comprised data from 9,815 separate properties across 135 of 139 Eircode routing key areas, but to avoid idiosyncrasies associated with small samples, we exclude Eircode routing key areas where there are less than 20 separate properties.^1^ Researchers wishing to utilise the publicly accessible dataset may wish to increase that cut-off value further. The final dataset, accessible at Curtis and Farrell [13], comprises data from 84 Eircode routing key areas, with a higher concentration of observations in the wider Dublin hinterland.

Table 1 reports a sample of the dataset covering period 6–8pm in November for approximately half of the Eircode routing key areas available in the dataset. Data related to Eqs. (1) and (2) are not reported in Table 1. The first row reports data for Eircode routing key A63, which is the north Wicklow area. The data is from 60 properties, with 5,476 separate observations for the 6–8pm period in November. During this two-hour slot, on average, heating systems are turned on 37 % of the time (across every day of November in the two year's of data), whereas the boiler is firing approximately 28 % of the time. As a practical illustration, a home might have its heating switched on for 8 hours and 53 minutes, possibly split across time the morning and in the evening. To maintain indoor temperatures at the setpoint level usually does not require the boiler to be firing continuously, instead it fires intermittently as necessary. In total, for this example, the boiler fires for 6 hours and 43 minutes. These two durations represent 37 % and 28 % of a 24 day. Properties in the sample rely on either gas or oil as their primary heating fuel source. Just over three-quarters of the 60 properties in A63 use gas as their heating fuel. Across the areas listed in Table 1, the mean percent of time the heating is switched on within an Eircode area varies from 26 % in E32 (Carrick-on-Suir area in Co. Tipperary) up to 56 % in D20 (includes Chapelizod, and Palmerstown in Dublin). The mean percent of time the boiler is firing is always lower and represents the time when fuel is being consumed.

**Table 1:** Sample of data for 6–8pm in November.

			Proportion	Heating on, %		Boiler firing, %	
Eircode	Hubids	Obs	gas fuelled	Mean	Std. Dev.	Mean	Std. Dev.
A63	60	5476	0.76	37.41	42.19	28.45	34.18
A82	45	3517	0.59	40.12	43.33	25.97	32.66
A85	42	3771	0.78	37.83	40.80	29.18	33.25
A92	162	14074	0.78	37.46	42.00	30.19	35.56
A96	82	7319	0.92	47.61	44.31	36.97	37.60
C15	131	11242	0.55	35.56	42.42	26.74	34.37
D03	62	5462	0.95	43.61	43.14	32.06	34.55
D04	22	2028	1.00	33.51	41.29	23.20	31.35
D07	92	7655	0.99	43.23	43.02	33.24	35.21
D08	57	4780	0.98	39.67	41.73	30.59	34.00
D11	253	21983	0.96	46.24	43.93	35.97	36.21
D12	211	19072	0.96	47.21	43.61	37.85	36.82
D15	341	30463	0.94	42.03	43.01	32.05	35.33
D16	129	11348	0.89	42.42	43.66	34.39	36.76
D20	66	6555	0.94	51.66	43.44	39.91	36.52
D22	410	38583	0.91	42.51	43.02	33.37	35.72
E32	34	2838	0.63	26.30	37.89	20.94	30.49
E41	50	4357	0.52	30.37	40.54	23.28	32.38
E91	47	3881	0.65	34.73	41.87	27.38	34.23
F23	39	3584	0.48	35.21	40.09	30.02	35.05
F28	27	2109	0.64	26.71	38.34	20.89	30.95
H91	92	9267	0.48	36.23	41.76	27.62	33.85
K34	29	2471	0.91	34.48	40.89	25.82	33.58
K45	41	3635	0.84	32.56	40.21	25.54	33.14
K67	175	15496	0.91	40.02	42.40	30.45	34.61
N39	20	1949	0.43	36.73	43.52	26.07	34.55
P51	38	3287	0.52	29.94	39.95	22.50	31.96
R14	61	5293	0.71	32.84	40.99	23.91	32.57
R35	42	3232	0.65	29.77	40.90	23.67	33.15
R51	42	3389	0.63	36.29	41.92	28.74	34.72
R95	130	10170	0.56	44.01	44.72	32.53	35.86
T23	48	4820	0.84	41.81	43.87	30.70	36.37
V92	24	2278	0.37	31.62	41.25	23.95	32.68
V95	84	7414	0.45	36.10	43.67	27.86	35.55
W23	230	20250	0.74	41.24	43.14	30.78	35.31
X35	38	3690	0.62	43.84	44.76	33.49	36.71
Y14	68	5842	0.52	37.28	42.53	28.71	34.57
Y25	131	10299	0.44	34.04	42.44	25.78	34.30
Y35	145	12091	0.42	33.43	41.47	25.66	34.02

**Fig. 1:**
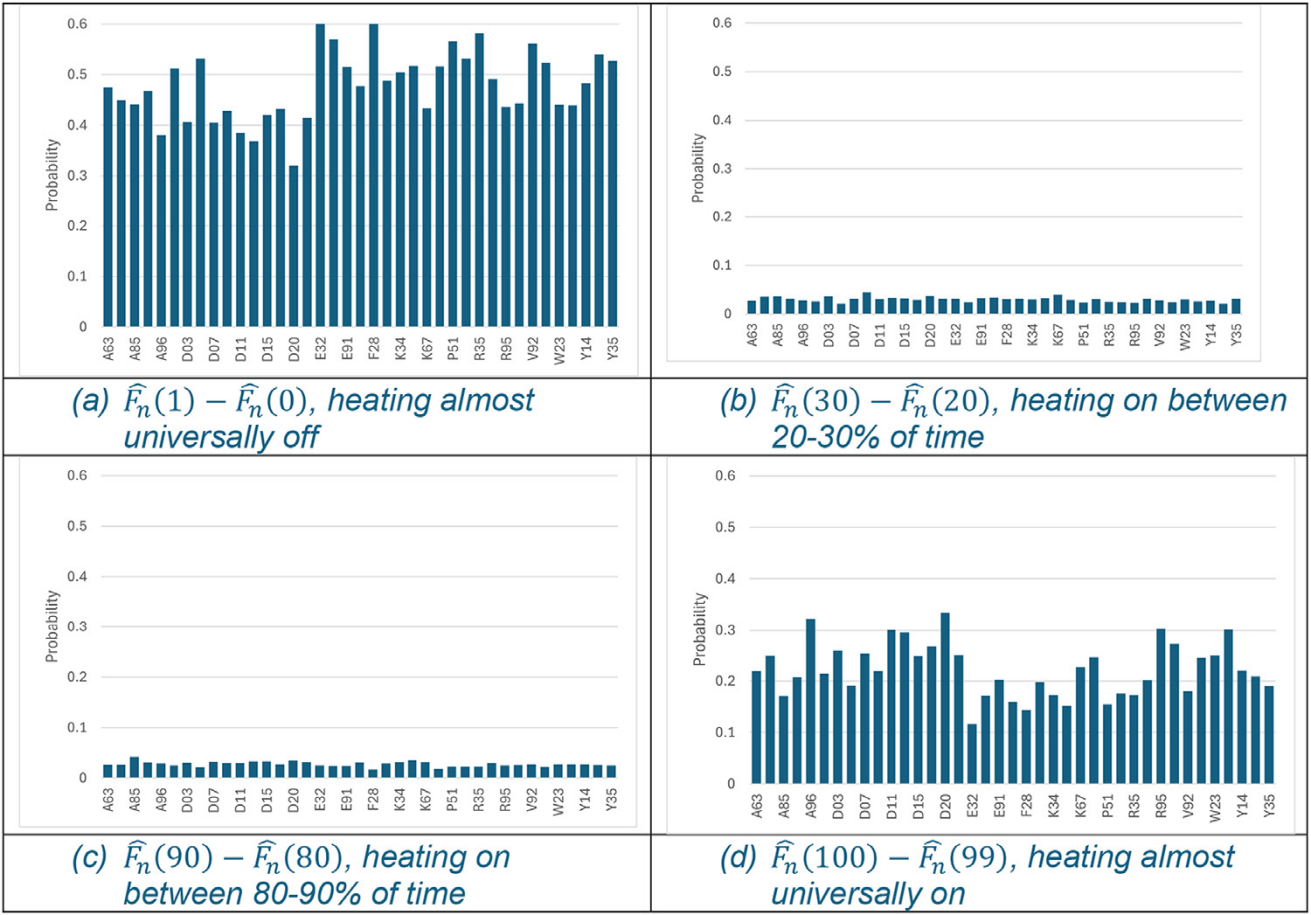
Probability heating turn on across selected Eircode routing keys for 6-8pm in November.

**Fig. 2:**
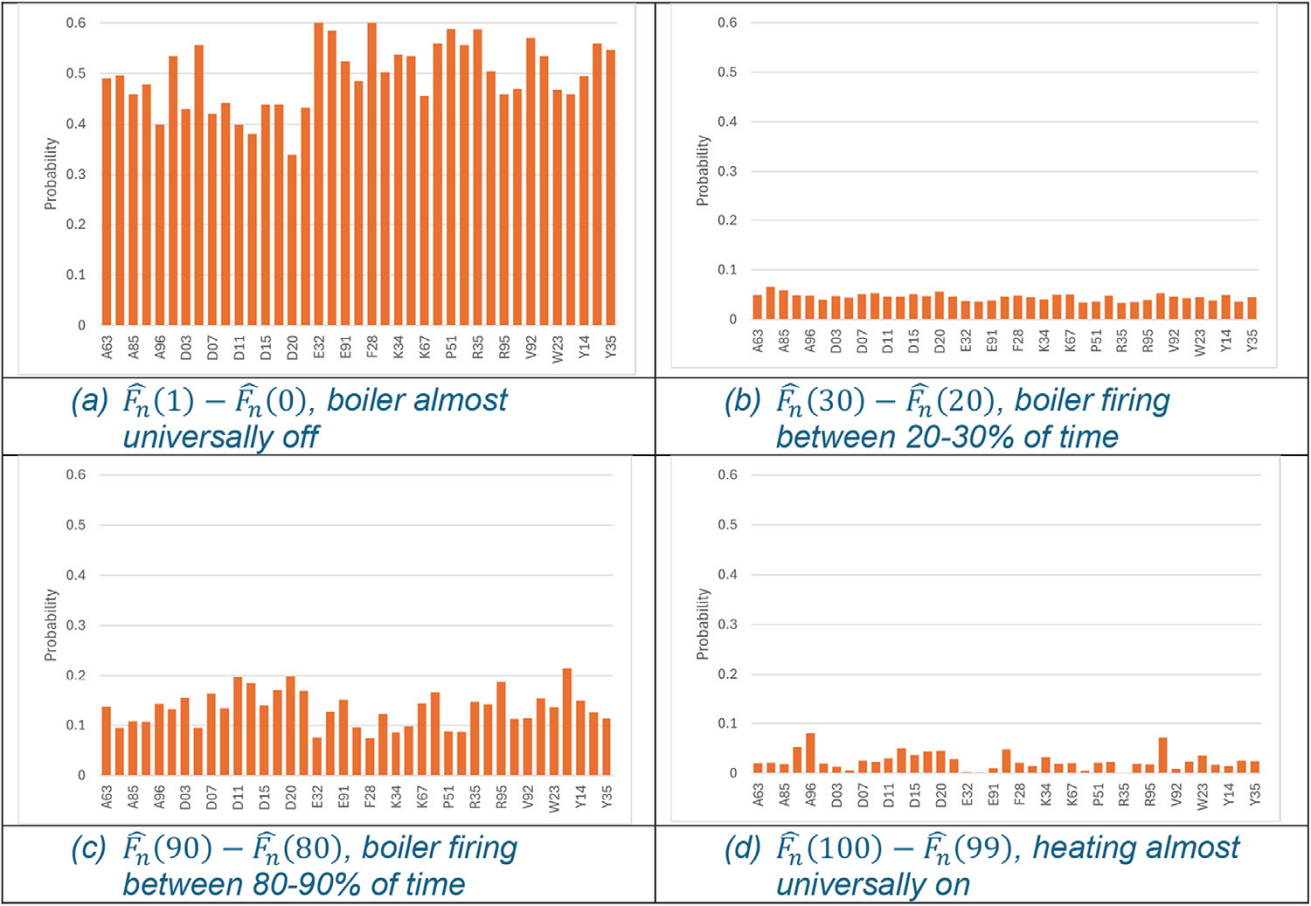
Probability boiler firing on across selected Eircode routing keys for 6–8pm in November.

Although Table 1 represents just a small subset of the entire dataset, it easily illustrates the inherent heterogeneity across households in heating profiles. On average, heating systems are turned on for twice as long in D20 as E32 during the 6–8pm period in November. The dataset provides no insight into what is driving this difference, whether it's related to occupancy, income, or energy efficiency. Fig. 1, Fig. 2 illustrate the heterogeneity graphically. Panel (a) in both figures shows how the mean of the number of properties with the heating/boiler turned off for the entirety of the specified 2-hour slot varies across Eircode areas. The bi-modal nature of the distributions is also evident. Across Eircode areas, on average, more than two-thirds of households have their heating either turned off (Fig. 1:a) or turned on (Fig. 1:d) for the entirety of the 2-hour slot. The bi-modal distribution of the boiler firing is somewhat different. The probability of households where the boiler is firing almost entirely for the 2-hour slot is relatively small, as illustrated in Fig. 2:(d) but the bi-modal feature is evident in Fig. 2:(c) where there is a notable up step in the probability that the boiler is firing between 80–90 % of the time. Overall, the data illustrates that there is considerable heterogeneity in energy use for heating across households, and that heterogeneity has a spatial dimension.

## 4 Experimental Design, Materials and Methods

The completed dataset is drawn from approximately 10,000 Irish homes, covering a period of 24 months from 1 October 2019 until 30 September 2021, and which are customers of energy services company Hub Controls Ltd. Hub Controls’ smart thermostat logs data approximately every three minutes related to indoor temperature and humidity, thermostat setpoints, and boiler operation among other parameters. Given the large number of house- holds, and the high frequency data logging, the total filesize for the raw dataset exceeds 915GB, which adds to the challenge of utilising the data.

The dataset contains no information about either occupants or property attributes. Meles et al. [11,12] drew information on property attributes from the Building Energy Rating (BER) database, where it was feasible, but in doing lost approximately 93 % of households from the data that they subsequently analysed. A BER rating encapsulates a range of information on the property's attributes but Meles et al. [11] find that there is more variability in energy efficiency performance within BER categories than across BER categories, and that a property's BER is not a good predictor of energy use. For those reasons, having research data based on a large N without property attribute data, may be preferable for some research purposes compared to a small N dataset with property attributes. This may be particularly relevant to investigating the impact on the electricity network.

The ambition for the dataset is to represent the variability of heating demand across households. We do this by Eircode routing key, for each of 12 two-hour slots (i.e., 00:00–02:00, 02:00–04:00, etc.), and 12 calendar months (i.e. Jan, Feb, etc.) reporting values for mean and standard deviation. For example, taking all data within the 2-hour slot 8–10pm, across the month of December, for all properties with routing key ‘A67’, the heating is turned on 32 % of the time, while boilers are firing 24 % of the time. While mean and standard deviation are useful information, the distribution is not a typical symmetrical bell shape. To fully reveal the variability in heating demand we calculate the empirical cumulative distribution function (ECDF) of the time the heating is switched on, and similarly for when the boiler is firing, as follows

(1) $$\hat{F_{n}}\left(t\right)=\frac{1}{n}\sum_{i=1}^{n}b1_{x_{i}}^{\mathrm{emh}}\leq t\;\forall \;e,m,h$$

where $1_{x_{i}}^{\mathrm{emh}}$ is a variable indicating when the heating is turned on (or the boiler is firing) for a given routing key e, month m, and 2-hour slot h [14]. $\hat{F_{n}}\left(t\right)\;is\;$the fraction of observations that are less than or equal to the specified value of *t*. In the dataset we report discrete increments of the ECDF as follows:

(2) $$\hat{F_{n}}\left(t_{0}\right)-\hat{F_{n}}\left(t_{1}\right)$$

This approach preserves information associated with the substantial proportion of properties where the heating/boiler is either turned on or off for the entirety of any particular 2-hour slot. It is the prevalence of households with their heating switched entirely on or entirely off for the duration of the 2-hour slots that underpins the bi-modal distribution referred earlier.

## Limitations

A limitation of the current dataset is that there is neither information about the condition of the properties (e.g., thermal efficiency) nor their occupants (e.g., thermal comfort preferences), which may vary substantially across households. Accordingly, the data aggregated to postal routing key areas incorporates the heterogeneity of heating behaviours reported as mean values. This has relevance to researchers interested in heating loads at a spatial scale but is less useful to understand the heterogeneity of individual homes heating loads.

## Ethics Statement

Authors affirm that they adhere to ethical guidelines for publishing. The present article does not include human subjects, animal experiments, or data obtained from social media platforms.

## CRediT Author Statement

**John Curtis:** Conceptualization, Methodology, Software, Writing - Original Draft. **Niall Farrell:** Conceptualization, Data Curation, Writing - Original Draft, Project administration.

## Data Availability

Mendeley DataIrish residential heating demand profiles (Original data).

## Footnotes

1 An Eircode comprises two parts, a Routing Key, and a Unique Identifier. The Routing Key identifies the principal postal town related to a property's address. The Routing Key characters are the same for all addresses within that postal area. The Unique Identifier is distinctive to an address and its geographic location. Our dataset contains only Routing Key information, so the granularity of our spatial identifier is at the postal area.
